# An undifferentiated cause of rhabdomyolysis: a case report

**DOI:** 10.1186/s12245-023-00507-y

**Published:** 2023-05-11

**Authors:** Pallavi Patil, Jennifer Davidson, Sundip Patel

**Affiliations:** 1 Cooper Medical School of Rowan University, Camden, NJ USA; 2 Department of Emergency Medicine, Cooper University Hospital, Camden, NJ USA

**Keywords:** Autoimmune myositis, Rhabdomyolysis, Case report

## Abstract

**Background:**

Rhabdomyolysis can occur secondary to infections, trauma, or myotoxic substances. Rhabdomyolysis secondary to autoimmune myositis occurs rarely. Distinguishing autoimmune rhabdomyolysis from rhabdomyolysis secondary to other causes is paramount in considering the long-term management of autoimmune rhabdomyolysis. It is further important to continue close follow-up and further testing to completely understand the extent of this disease as diagnoses may be ever-changing.

**Case presentation:**

A previously healthy female presented to the hospital with myalgias and myoglobinuria following a respiratory infection treated with azithromycin and promethazine. Labs demonstrating elevated creatine kinase (CK) prompted treatment for rhabdomyolysis and rheumatology consultation. The patient was given 3 l of intravenous (IV) 0.9% sodium chloride in the Emergency Department. Upon admission, the patient was placed on a continuous IV drip of 0.9% sodium chloride running at 300 cc/hour for all 8 days of her hospital admission. The rheumatology autoantibody panel pointed towards autoimmune myositis as a potential cause of her rhabdomyolysis. The patient was discharged to follow up with rheumatology for further testing.

**Conclusion:**

Autoimmune myositis, although less common than other etiologies of rhabdomyolysis, is important to consider as the long-term management of autoimmune myositis includes the use of immunosuppressants, antimalarials, or IV immunoglobulins, which may be inappropriate for other etiologies of rhabdomyolysis.

## Background

Rhabdomyolysis, or the destruction of striated muscle cells, involves loss of muscle cell integrity leading to the leakage of cell contents into the bloodstream [1]. Damage to the muscle cells can occur through a variety of mechanisms. Viral infections, through direct mechanisms of viral invasion or through indirect mechanisms involving toxin generation, have been shown to commonly cause muscle damage. Influenza, Parainfluenza, Adenovirus, Herpes Simplex, and now COVID-19, are all viruses that have been implicated in rhabdomyolysis [2, 3]. Bacterial infections, through similar mechanisms, have also been tied to rhabdomyolysis with the most common bacterial agents being Streptococcus species, Salmonella, and Francisella tularensis [2]. Outside of viral and bacterial infections, trauma via crush injuries is a common cause of rhabdomyolysis. Substances that have been seen to cause rhabdomyolysis include medications with direct myotoxicity such as erythromycin, cyclosporine, corticosteroids, and colchicine as well as substances which lead to indirect damage such as alcohol, cocaine, and amphetamine [1].

No matter the initiating factor, rhabdomyolysis, through direct or toxin-mediated actions, involves the common pathway of muscle cell breakdown, leakage of cell contents, and ultimately presents with the classic triad of myalgias, generalized weakness, and tea-colored urine. A diagnosis of rhabdomyolysis can then be further supported through lab studies demonstrating an elevated serum creatine kinase, more than five times the upper limit of normal (> 975 IU/L) [4], and positive urine myoglobin [1, 5]. The exposure of the kidney to increased levels of myoglobin leads to the characteristic kidney injury seen in rhabdomyolysis which may progress to acute renal failure. A missed diagnosis of rhabdomyolysis could lead to the patient requiring life-long dialysis treatments.

When the common causes of rhabdomyolysis have been ruled out, one must start considering uncommon causes such as autoimmune. This rare cause of rhabdomyolysis involves an aberrant response of the body’s immune system which leads to an attack of the body’s own tissues and can be seen as dermatomyositis, polymyositis, necrotizing autoimmune myositis, and inclusion body myositis [6]. In this case report, we present the case of a patient presenting with rhabdomyolysis which was initially thought to be due to a viral illness or medications and upon discharge was thought to be a result of an autoimmune cause. After close follow-up spanning 4 years, the patient’s rhabdomyolysis was ultimately deemed undifferentiated as autoimmune was not ruled out but considered less likely.

## Case presentation

A 41-year-old female with a prior medical history of hypertension, menorrhagia requiring blood transfusions, and polycystic ovarian syndrome presented to the Emergency Department (ED) with acute onset of myalgias. The patient’s medications included amlodipine 5 mg for blood pressure control, ibuprofen 600 mg for analgesia, and tranexamic acid 650 mg for heavy menstrual bleeding. One week prior to the onset of her diffuse muscle pain, she presented to an urgent care with cough, chills, and sore throat. At the urgent care, a chest X-ray was performed which demonstrated no consolidations or any other acute abnormalities. She was diagnosed with bronchitis and prescribed promethazine and a 5-day course of azithromycin (500 mg of azithromycin on day 1 and then 250 mg each day for 4 more days) and noted symptom improvement. However, 3 days prior to her arrival at the ED, she began to experience diffuse lower extremity muscle aches which she described as “spasms.” She further endorsed dark urine. On the day of ED arrival, she visited her primary care provider who was concerned about jaundice on her physical examination and sent the patient in for further evaluation.

On arrival at the ED, the patient had a blood pressure of 141/91 mmHg, a pulse of 77 beats per minute, a temperature of 98.3°F (36.8 °C), a respiratory rate of 18, and a SpO_2_ of 98%. Physical examination demonstrated a patient with jaundice who was non-toxic in appearance and with no other remarkable physical findings. Classic cutaneous manifestations of dermatomyositis including Heliotrope rash, Gattron’s sign (raised lesions overlying extensor tendons of the extremities), Gattron’s papules (raised lesions overlying hand joints), or Shawl sign [7] (rash distributed where a shawl normally covers skin on the neck, upper back, upper chest, and shoulders) were not seen. In the ED, the patient’s creatine kinase (CK) was found to be elevated at 129,830 units/liter (U/L) (reference range: 32–182 U/L). In addition, a urinalysis demonstrated myoglobinuria. While in the Emergency Department, she was given 3 l of intravenous (IV) 0.9% sodium chloride. Upon admission, the patient was placed on a continuous IV drip of 0.9% sodium chloride running at 300 cc/hour for her entire hospital stay. She was additionally offered morphine for analgesia and anti-tussive medication for her cough. During her admission, the patient’s CK peaked at 230,260 U/L. The patient’s kidney function was also monitored. When she first arrived, her BUN was 9 mg/dL (reference range: 6–24 mg/dL) and creatinine was 0.55 mg/dL (reference range: 0.57–1.00 mg/dL). By day seven, prior to discharge, her BUN decreased to 6 mg/dL and her creatinine was 0.34 mg/dL. Her serum sodium, potassium, and chloride remained within normal ranges throughout her stay (Table 1). Upon arrival, the patient’s liver enzymes were elevated with an AST of 601 U/L (reference range: 10–35 U/L) and an ALT of 135 U/L (reference range: 6–45 U/L). Her liver enzymes peaked on day 4 of her hospital stay with an AST of 986 U/L and an ALT of 259 U/L before trending down to an AST of 334 U/L and an ALT of 238 U/L prior to discharge. Her viral panel, which tested hepatitis A IGM antibody, hepatitis B surface antigen, hepatitis B core antibody IGM, HCV antibody screen, HIV, CMV, EBV, influenza, adenovirus, parainfluenza, and RSV, was negative. While in the hospital, an MRI of her thighs was done which showed intramuscular edema to the following muscle groups on her left side (gluteus maximums, sartorius, vastus medialis, rectus femoris, biceps femoris, semitendinosus, gracilis muscles) and right side (vastus lateralis, rectus femoris, vastus medialis, sartorius, gracilis, semitendinosus, semimembranosus, biceps femoris) indicative of bilateral thigh myositis. Rheumatology was consulted while the patient was in the hospital. They initially reported that the likely cause of her rhabdomyolysis was viral myositis, however continued to work up different etiologies of rhabdomyolysis by ordering a muscle biopsy, ANA IFA with reflex, ESR/CRP, myositis panel, C3/C4, SSA/SSB, RF, and aldolase (Table 2). The patient’s muscle biopsy presented without significant histopathology and the myositis-specific autoimmune antibody panel showed positive TIF-1y Ab indicating autoimmune to be a more likely cause of her rhabdomyolysis compared to a viral etiology.

**Table 1:** Creatinine and electrolyte values upon hospital admission and at hospital discharge

	**Day of admission**	**Prior to discharge**
*Creatinine (0.6–1.20 mg/dL)*	0.55	0.34
*Glucose (70–110 mg/dL)*	93	96
*Sodium (135–145 mmol/L)*	140	137
*Potassium (3.5–5.0 mmol/L)*	3.5	3.9
*Chloride (96–108 mmol/L)*	100	102
*Calcium (8.5–10.5 mg/dL)*	8.8	7.8

**Table 2:** Autoantibody panel results during the patient’s inpatient hospital stay

	**Patient values**	**Reference range and units**
*ANA screen, IFA*	Negative	Negative
*ESR*	17	0–20 mm/h
*CRP*	0.57	< 0.50 mg/dL
*JO-1 Ab*	< 11	< 11 SI
*PL-7 Ab*	< 11	< 11 SI
*PL-12 Ab*	< 11	< 11 SI
*EJ Ab*	< 11	< 11 SI
*OJ Ab*	< 11	< 11 SI
*SRP Ab*	< 11	< 11 SI
*MI-2 Alpha Ab*	< 11	< 11 SI
*MI-2 Beta Ab*	< 11	< 11 SI
*MDA-5 Ab*	< 11	< 11 SI
*TIF-1y Ab*	20	< 11 SI
*NXP-2 Ab*	< 11	< 11 SI
*C3 complement*	136	86–184 mg/dL
*C4 complement*	45	20–59 mg/dL
*SSA Ab*	< 1.0 NEG	< 1.0 NEG
*SSB Ab*	< 1.0 NEG	< 1.0 NEG
*Aldolase*	< 1.0	< OR = 8.1 U/L
*Rheumatoid factor*	Negative	Negative

**Fig. 1:**
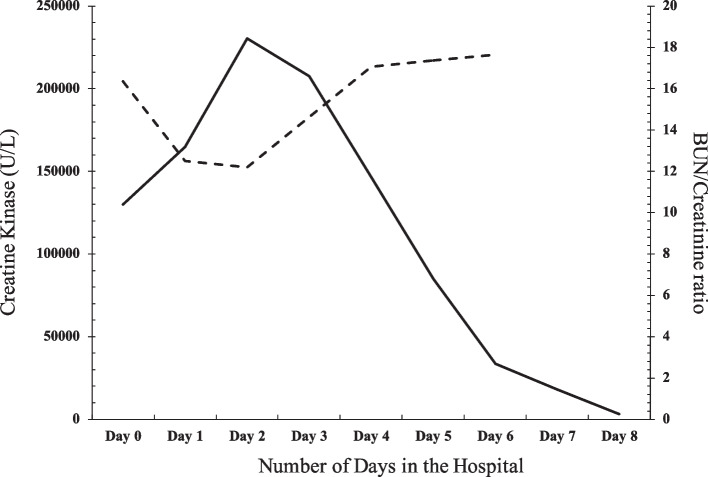
Progression of creatine kinase levels in a 41-year-old female during hospital admission for rhabdomyolysis (solid line). Progression of BUN/creatinine ratio as an indicator of kidney function (dotted line). During this period, the patient was being treated with normal saline at 300 cc/hour. Day 0 represents the day the patient presented to the Emergency Department, and day 8 represents the day the patient was released

The patient’s condition improved rapidly and on day 8 of her hospital admission, her CK had decreased to 18,000 (Fig. 1). Prior to discharge her CK level was 3280U/L. She was instructed to follow up with rheumatology for close monitoring and upon follow-up, and her creatine kinase levels had decreased to 233 U/L. Since her hospitalization, her creatine kinase levels have ranged from 205 to 399 U/L. The patient continues to follow up with rheumatology outpatient and has remained clinically stable since discharge. In the setting of her CK remaining only mildly elevated, the decision was made to hold off on further treatment. Consideration was given to a course of azathioprine or methotrexate if the CK had remained markedly elevated but was withheld due to good response from inpatient treatment with aggressive fluids. In the setting of a positive TIF-1y ab, the patient underwent a colonoscopy, mammogram, PAP smear, CT chest, abdomen, and pelvis to screen for an associated malignancy. Thus far, all malignancy screenings have been negative. Due to the patient’s decreased CK levels, negative malignancy work-up and stable condition, rheumatology recently deemed this patient’s case of rhabdomyolysis to be undifferentiated.

## Discussion

Rhabdomyolysis, although most commonly reported to be caused by trauma, drugs, and infections in the adult population, may also be caused by less common causes such as the progression of autoimmune myositis [8]. Autoimmune forms of myositis are rare and include dermatomyositis, polymyositis, necrotizing autoimmune myositis, and inclusion body myositis. Autoimmune myositis results from the body’s natural immune system responding aberrantly and inappropriately attacking the body’s own tissues. Autoimmune attack of tissues may involve cytotoxic T cells, macrophages, autoantibodies, and/or complement activity depending on the disorder [6]. Similar to other forms of rhabdomyolysis, the presentation of autoimmune rhabdomyolysis is clinically characterized by myalgias, muscle weakness, and muscle swelling with possible dark urine. Laboratory findings in autoimmune rhabdomyolysis will demonstrate an elevation of creatine kinase as well as elevations in aldolase, myoglobin, lactate dehydrogenase, aspartate aminotransferase, and alanine aminotransferaseas can be seen in other causes of rhabdomyolysis. However, the most specific serum tests for autoimmune rhabdomyolysis are antibodies such as anti-Ro, anti-La, anti-PM-Scl, TIF-γ, TIF-α, and anti-nuclear matrix protein 2 (NXP2). In addition, a muscle biopsy is important in the workup for autoimmune rhabdomyolysis; however, the biopsy should be performed after recovery from the acute episode as there is a concern that muscle necrosis could cover underlying pathology. Muscle biopsy of autoimmune rhabdomyolysis classically demonstrates lymphocytic infiltrate and may demonstrate perifascicular atrophy. Of note, patients with immune-mediated necrotizing myopathy may present with necrosis on muscle biopsy without the presence of inflammatory cells. In patients with autoimmune myositis with negative muscle biopsies, testing for autoantibodies (i.e., anti-signal recognition particle or anti-HMG-CoA reductase autoantibodies) may assist in making the correct diagnosis [9]. MRI can aid in the workup of rhabdomyolysis by demonstrating muscle necrosis and inflammation, revealing active muscle involvement [10].

Acute viral myositis is characterized by muscle inflammation secondary to a viral infection. The most common causes of viral myositis include influenza and enteroviruses with symptoms starting a few days following the onset of the infection. The patient presented with rhabdomyolysis 11 days after her fever began, prompting the consideration of viral myositis. However, while admitted to the hospital, the patient had testing which revealed a negative viral panel even though her CK levels remained elevated. The negative viral panel along with her increasing CK levels prompted the physicians to order an autoimmune panel.

Over 150 drugs, including azithromycin, have been implicated in drug-induced myositis. Azithromycin has been reported to mildly inhibit cytochrome P450 (CYP) 3A4 and has further been reported to cause rhabdomyolysis particularly when given with a statin [11]. A differential diagnosis of drug-induced myositis was discussed briefly secondary to the patient’s recent completion of azithromycin. However, this differential was ruled out as the CK levels continued to rise despite the discontinuation of the azithromycin. Trauma-induced rhabdomyolysis was not considered as the patient did not have a crush injury or endured intense muscular exercises which could also lead to rhabdomyolysis [12].

Although viral infections, drug reactions, and trauma are some of the main causes of rhabdomyolysis, it is important to consider the less common causes such as autoimmune myositis. Initial treatment of rhabdomyolysis is similar despite the inciting cause. The first-line treatment includes intervention aimed at preventing acute kidney injury through the use of hydration with IV fluids. IV hydration with lactated Ringer’s or saline is recommended within 6 h of admission as the chance of acute kidney injury increases with delayed initiation [2, 13]. Clear evidence for the use of sodium bicarbonate, to alkalinize urine, and diuretics, to minimize cell injury, is lacking. Treatment of rhabdomyolysis then differs based on pathogenesis after this first line of care [14]. Viral rhabdomyolysis and trauma-induced rhabdomyolysis treatment end here with supportive measures. Bacterial rhabdomyolysis treatment will include the addition of appropriate antibiotics. Autoimmune rhabdomyolysis treatment requires further management with the use of high-dose IV steroids. Management of autoimmune myositis after hospital discharge may include immunosuppressants such as methotrexate, azathioprine, cyclosporine, tacrolimus, and mycophenolate mofetil. Antimalarials, such as hydroxychloroquine and chloroquine, may also be effective in treating dermatomyositis. Although remission rates may vary by individual, antimalarials are generally safe and well tolerated. Intravenous immunoglobulin can be considered for treatment-refractory autoimmune myositis [7]. After recovery, for all rhabdomyolysis cases, physical therapy may be recommended to improve muscle function and regain strength.

Autoimmune rhabdomyolysis, although rarely encountered, is a vital differential diagnosis to consider when encountering patients in the ED. While initial treatment is similar for all cases of rhabdomyolysis, long-term management and prevention of recurrence will differ. It is further vital to encourage close monitoring with rheumatology and periodic lab work. In this case, our patient remains an undifferentiated case of rhabdomyolysis, proving the complexity of finding the cause of rhabdomyolysis in certain cases. It is important to note that autoimmune myositis has not been completely ruled out and further follow-up and monitoring is needed.

## Conclusion

There are several etiologies for rhabdomyolysis, including viral, medication-induced, and autoimmune. Morbidity and mortality of rhabdomyolysis are closely linked to an accurate diagnosis and appropriate timely treatment of the underlying etiology. In this case, the patient presented with muscle weakness, pain, myoglobinuria, and elevated creatine kinase levels indicating rhabdomyolysis. While viral myositis and medication-inducted myositis were initially suspected, further testing and an extensive myositis panel pointed to an autoimmune etiology.

The patient’s improved CK levels and benign muscle biopsy was an indication to continue monitoring her without any medications, However, if her CK levels had continued to rise, the patient would have been treated with azathioprine or methotrexate. Rheumatology continues to work on differentiating the etiology of her disease state through close follow-up of her CK levels allowing for the prevention of relapse and consideration for future immunosuppression.

## Data Availability

Data sharing is not applicable to this article as no datasets were generated or analyzed during the current study.
